# Epidemiology of Musculoskeletal Injuries in Frisbee Athletes and Associated Risk Factors: World Beach Ultimate Championship

**DOI:** 10.3390/ijerph23050616

**Published:** 2026-05-07

**Authors:** Beatriz Minghelli, Vera Lúcia Ramos Guerreiro, Rodrigo Miguel Coelho Luz, Bruna Raquel Ferreira Rodrigues, Miguel Tomé Carminho

**Affiliations:** 1 Insight: Piaget Research Center for Ecological Human Development, 1950-157 Lisboa, Portugal; 2 Instituto Piaget de Silves, 8300-025 Silves, Portugal; 61502@ipiaget.pt (V.L.R.G.); 2022111563@ipiaget.pt (R.M.C.L.); 2022113681@ipiaget.pt (B.R.F.R.); 2022107724@ipiaget.pt (M.T.C.)

**Keywords:** athletic injuries, epidemiology, risk factors, sports, musculoskeletal diseases, prevalence, incidence, cross-sectional studies, athletes

## Abstract

**Highlights:**

**Public health relevance—How does this work relate to a public health issue?**

- Musculoskeletal injuries are common among frisbee athletes, a globally growing sport, representing an emerging public health concern in the sports context.
- The high prevalence of injuries, particularly in the lower limbs, may impact quality of life, sports participation, and impose a burden on healthcare systems.

**Public health significance—Why is this work of significance to public health?**

- This study provides novel epidemiological data on injuries among international-level frisbee athletes, helping to fill gaps in the scientific literature.
- The identification of risk factors, such as longer practice duration, supports the development of targeted and more effective preventive strategies.

**Public health implications—What are the key implications or messages for practitioners, policy makers and/or researchers in public health?**

- There is a need to develop and implement injury prevention programmes focusing on load management, neuromuscular training, and safe change-of-direction techniques.
- The findings can inform policies and guidelines to promote safety in emerging sports and guide future research on injury prevention and rehabilitation.

**Abstract:**

Frisbee has gained increasing popularity and is characterized by high-intensity running, rapid changes in direction, jumping, and indirect physical contact, exposing players to a risk of injury. This study aimed to determine the epidemiology of musculoskeletal injuries in frisbee athletes who participated in the World Beach Ultimate Championship in Portugal, and the risk factors associated with these injuries. The sample included 484 athletes aged 18–64 years, of whom 275 (56.8%) were male. Data was collected using a digital questionnaire. Across their frisbee practice, 391 (80.8%) athletes reported at least one injury, totalling 1685 injuries. A total of 49 (10.1%) athletes reported an injury in the last 7 days. Over the past 12 months, 211 (43.6%) athletes sustained injuries, totalling 358 cases. The injury proportion was 0.44 (95% CI: 39.2–48.0), and the injury rate was 0.67 injuries per 1000 h of training. The most frequent injuries were muscle strains (18.86%) and sprains (13.43%), mainly affecting the ankle (62; 17.71%) and knee (54; 15.43%). Repetitive movements (84; 22.11%) and changes in direction (62; 16.32%) were the most common mechanisms. Athletes with equal or more than 11 years of practice had a higher injury risk (OR = 1.84; 95% CI: 1.16–2.91; *p* = 0.009). Frisbee athletes present a considerable risk of injuries. Preventive strategies are recommended.

## 1. Introduction

The World Flying Disc Federation (WFDF) governs sports played with a flying disc, including Ultimate, Beach Ultimate, Disc Golf, Freestyle, and Guts [1]. Among these, Ultimate and its beach variant have experienced substantial global growth, with increasing participation and international competition [1,2]. Beach Ultimate is played on sand with smaller teams and field dimensions, resulting in distinct physical and mechanical demands compared to its grass counterpart [1].

The first Beach Ultimate tournament took place in 1986 in Texas. By the end of 2009, the sport was already practiced in over 25 countries with over 100 tournaments worldwide. The WFDF recorded an annual player increase rate of 11% over the last decade, highlighting the sport’s global expansion, with over 170,000 active athletes worldwide [1]. Frisbee is currently potentially eligible for future inclusion in the Olympic Games, a milestone resulting from the sport’s global growth. Nowadays, in the Portuguese Association of Ultimate and Disc Sports (APUDD) [3] there are currently 10 Frisbee teams, spread across the country.

Frisbee sports are characterized by high-intensity intermittent efforts, including sprinting, rapid changes in direction, jumping, and throwing actions. Although classified as a non-contact sport, the dynamic nature of play involves frequent contests for disc possession and incidental contact between players. These characteristics, combined with high biomechanical and physiological demands, may increase the risk of musculoskeletal injuries [4,5,6,7].

Sports-related injuries can negatively impact athlete performance, limit participation, and, in some cases, lead to long-term health consequences, highlighting the importance of injury prevention strategies [2].

In sports injury research, risk factors are commonly categorized as intrinsic (e.g., age, sex, previous injury history, and physical characteristics) and extrinsic (e.g., training load, playing surface, and environmental conditions). Sport injuries result from the interaction between these factors. Previous studies in team sports have consistently identified variables such as prior injury, training exposure, and playing conditions as important contributors to injury risk [4,5,6,7]. However, due to the specific demands of frisbee sports—particularly in beach environments, where unstable surfaces and altered movement patterns are present—the relative contribution of these factors may differ. Despite this, there is a lack of studies systematically examining these variables in Beach Ultimate athletes.

However, despite the rapid growth of frisbee sports, epidemiological data on injury patterns—particularly in Beach Ultimate—remain limited. Existing studies are scarce and often lack standardized injury definitions or exposure-based metrics, restricting comparisons across populations and competitive contexts.

Therefore, the aim of this study was to investigate the epidemiology of musculoskeletal injuries among athletes participating in the World Beach Ultimate Championship held in Portimão, Portugal. Specifically, this study sought to characterize injury prevalence and incidence, as well as to describe the most frequent injury types, locations, mechanisms, and associated risk factors.

## 2. Materials and Methods

The study consisted of a cross-sectional, descriptive-correlational study, approved by the KinesioLab–Research Unit in the Human Movement research centre.

This research was conducted in accordance with the Declaration of Helsinki. To participate in the study, all athletes signed an informed consent form, in which they declared their agreement to participate after the nature and objectives of the investigation had been explained to them. It was also clarified that they could withdraw from the study at any time, without the need for justification and without any form of prejudice. The data collected was used exclusively for scientific research purposes.

All reports followed the guidelines of the consensus statement Strengthening the Reporting of Observational Studies in Epidemiology (STROBE) Extension for Sports Injury and Illness Surveillance (STROBE-SIIS).

Population

The population consisted of 2277 Frisbee athletes who participated in the World Beach Ultimate Championships (WBUC) held at Praia da Rocha, in Portimão, southern Portugal, between 16 and 22 November 2025. The WBUC involved the participation of athletes from 38 countries.

The inclusion criteria were cumulative: athletes of any sex, aged 18 years or older, of any nationality, who voluntarily agreed to participate in the study, and who had practiced the sport for at least 12 months with a minimum training frequency of twice a week.

The sample size was determined using an estimated mean injury prevalence of 50% and assuming a 5% margin of error, with a 97% confidence interval (CI). A higher confidence level than the conventional 95% was selected to increase the precision of the estimates and to account for potential variability in injury reporting. Based on these assumptions, the minimum sample size was 391 athletes.

Measurement Instrument

The measurement instrument used was a digital questionnaire consisting of multiple-choice and open-ended questions.

The questionnaire was administered in the form of an interview by fourth-year Physiotherapy students from the School of Health Jean Piaget of Algarve-Piaget of Silves. Additionally, the questionnaire link was made available to the teams so that their athletes could respond. The sampling method was non-probabilistic, as participants were selected based on their availability and accessibility at the time of data collection.

The questionnaire was adapted from the study by Minghelli et al. [8], while the specific Frisbee-related questions were developed by the researchers. The questionnaire consisted of dichotomous (yes/no) items and categorical classification of injuries. Although no formal psychometric validation (e.g., construct validity or internal consistency analysis such as Cronbach’s alpha) was performed, the instrument underwent a rigorous development process. Content validity was ensured through evaluation by an expert panel of three experts which assessed the relevance, clarity, and comprehensiveness of the items: a PhD in Public Health specializing in epidemiology, who is also a researcher and physiotherapist; a PhD in Sports, Health and Exercise Sciences, who is also an exercise physiologist, President of the Ultimate Portimão Players Club, a Frisbee coach for 18 years, and an athlete for 22 years; and a Frisbee coach of 13 years, who had been an athlete for 21 years and is Vice President of Training at the Portuguese Association of Ultimate and Disc Sports (APUDD) and a member of the Ultimate Portimão Players Club.

Prior to the final application of the questionnaire, a pre-test was conducted with 16 athletes with similar characteristics to the study’s target population. This procedure aimed to evaluate the clarity, relevance and comprehensibility of the items, as well as to identify any potential ambiguities, interpretation difficulties or structural issues within the data collection instrument. Additionally, the pre-test allowed for an estimation of the average time required to complete the questionnaire and an assessment of the adequacy of the question sequence and organization. No changes to the questionnaire were necessary. The average completion time during the pre-test was 5 min and 30 s.

Given the nature of the instrument, which was designed primarily for descriptive classification rather than for the measurement of latent constructs, traditional reliability indices were not considered appropriate. Nevertheless, these procedures were implemented to strengthen the methodological rigour of the instrument and to ensure its suitability for the study objectives.

The questionnaire was divided into three sections: sociodemographic characteristics, aspects related to sports practice, and injury occurrence.

Injury was defined in line with the principles of the IOC consensus statement on injury surveillance in sports. Specifically, an injury was defined as any physical complaint resulting from Frisbee participation that resulted in (i) time loss from training or competition, (ii) a reduction or modification of normal training activities, or (iii) the need for medical attention. This broader definition was adopted to ensure comprehensive capture of both time loss and non-time-loss injuries.

Statistical Analysis

Data analysis was performed using the Statistical Package for the Social Sciences Statistics (IBM SPSS Statistics) software, version 30.0.

The analysis included descriptive statistics, utilizing measures of dispersion, central tendency, relative frequency and absolute frequency, as well as inferential statistics.

The proportion of injured athletes was determined by dividing the number of participants who reported at least one injury in the previous 6 months by the total number of athletes included in the study. The injury rate was calculated as the total number of injuries relative to the total exposure time, expressed per 1000 h of risk. Exposure time was estimated by multiplying the average weekly training duration (in hours) by the weekly training frequency, which was subsequently extrapolated to a 6-month period (26 weeks).

Additionally, the mean number of injuries per athlete was obtained by dividing the total number of recorded injuries by the overall sample size. The average number of injuries among injured athletes was calculated by dividing the total number of injuries by the number of participants who sustained at least one injury.

To examine the association between independent variables and the occurrence of injury within the previous 12 months, logistic regression analysis was conducted. Initially, univariable logistic regression models were performed for each independent variable. Subsequently, a multivariable logistic regression model was constructed using the Enter method, in which all variables of interest were included simultaneously, regardless of their significance in the univariable analysis. Categorical variables were entered into the model using appropriate reference categories.

The results of the logistic regression analyses were expressed as odds ratios (OR) with 95% confidence intervals (95% CI).

Multicollinearity among independent variables was assessed using variance inflation factors (VIF), tolerance values, and condition indices. VIF values ranged from 1.01 to 1.22, and tolerance values ranged from 0.82 to 0.99, indicating no evidence of multicollinearity.

Model performance was evaluated using the Omnibus test of model coefficients, pseudo R-squared measures (Cox & Snell and Nagelkerke), and the Hosmer–Lemeshow goodness-of-fit test. The agreement between observed and predicted values was also examined through the Hosmer–Lemeshow contingency table.

Statistical significance was assessed using the Wald test, and a *p*-value < 0.05 was considered statistically significant.

## 3. Results

Six hundred and forty-three athletes agreed to participate in the study; however, 44 (6.84%) did not have at least one year of experience in the sport, and 117 (18.19%) did not have a weekly training frequency of at least twice a week in the last 12 months. As a result, the sample consisted of 484 frisbee athletes aged between 18 and 64 years (33.88 ± 8.94 years), including 275 (56.8%) males and 209 (43.2%) females. Body weight ranged from 40 to 125 kg (70.58 ± 10.99); height ranged from 1.40 to 2.00 m (1.74 ± 0.11); and body mass index (BMI) ranged from 17.94 to 34.63 kg/m^2^ (23.08 ± 2.16). According to BMI classification, 3 (0.6%) athletes were classified as underweight, 401 (82.9%) had normal weight, 76 (15.7%) were overweight, and 4 (0.8%) were classified as obese.

Regarding limb dominance, the right side was the most common (425; 87.8%), while 59 (12.2%) athletes reported left-side dominance.

In this sample, the most common nationality was Canadian, represented by 43 (8.9%) athletes, followed by American (42; 8.7%), Australian and Spanish (39; 8.1% each), English (29; 6.0%), German (28; 5.8%), Italian (24; 5.0%), Portuguese (22; 4.5%), Belgian and French (19; 3.9% each), Indian (18; 3.7%), Swiss and Austrian (12; 2.5% each), Dutch and Japanese (11; 2.3% each), Finnish and Singaporean (9; 1.9% each), Colombian (8; 1.7%), Czech, Filipino, Latvian, and Lithuanian (7; 1.4% each), Danish, Slovak, Mexican, Turkish, and Ukrainian (6; 1.2% each), Lebanese, South African, and Swedish (4; 0.8% each), Chinese, Irish, Israeli, Polish, and Venezuelan (3; 0.6% each), and Estonian and Qatari (1; 0.2% each). Three (0.6%) athletes reported other nationalities but did not specify them.

**Figure 1:**
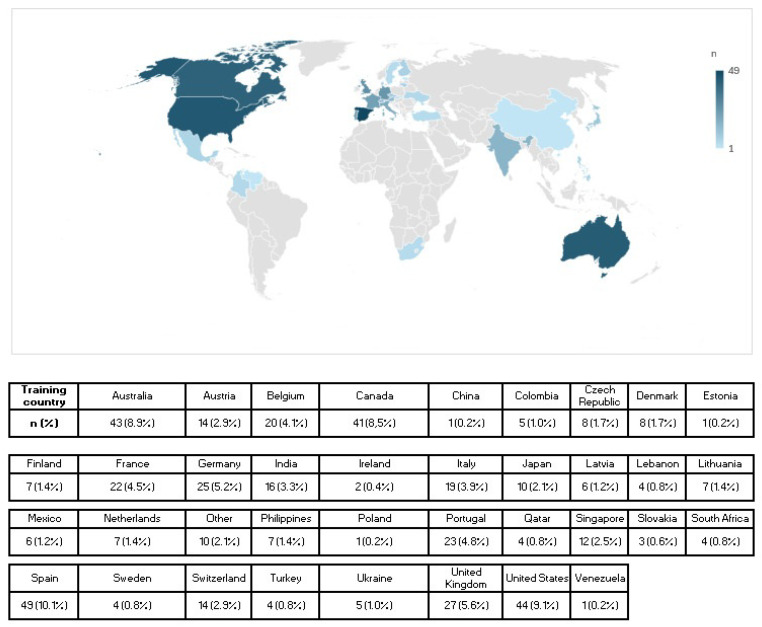
Countries where the athletes trained.

Figure 1 illustrates the countries where the athletes included in the study were training.

Table 1 presents the sample characteristics related to variables associated with frisbee practice.

Over the athletes’ entire frisbee practice history, 80.8% (n = 391) reported at least one injury, while 19.2% (n = 93) reported no injuries, yielding a total of 1685 injuries. In the 7 days preceding data collection, the injury prevalence was 10.1% (n = 49), whereas 89.9% (n = 435) reported no injuries. Over the previous 12 months, 43.6% of athletes (n = 211) reported at least one injury, corresponding to a total of 358 injuries, while 56.4% (n = 273) remained injury-free during this period. As only the three most severe injuries per athlete were further characterized, subsequent analyses were based on 350 injuries.

The 12-month injury prevalence was 0.44 (95% CI: 39.5–48.0). The injury incidence rate was 0.67 injuries per 1000 h of frisbee exposure. On average, athletes sustained 0.74 injuries per athlete, and 0.91 injuries per injured athlete.

Among athletes who reported at least one injury in the previous 12 months (n = 211), 47.4% (n = 100) experienced abrasions. Of these, 61% (n = 61) reported that abrasions occurred rarely, whereas 39% (n = 39) reported them occurring regularly.

Table 2 presents the association between injury type and anatomical location over the past 12 months.

**Figure 2:**
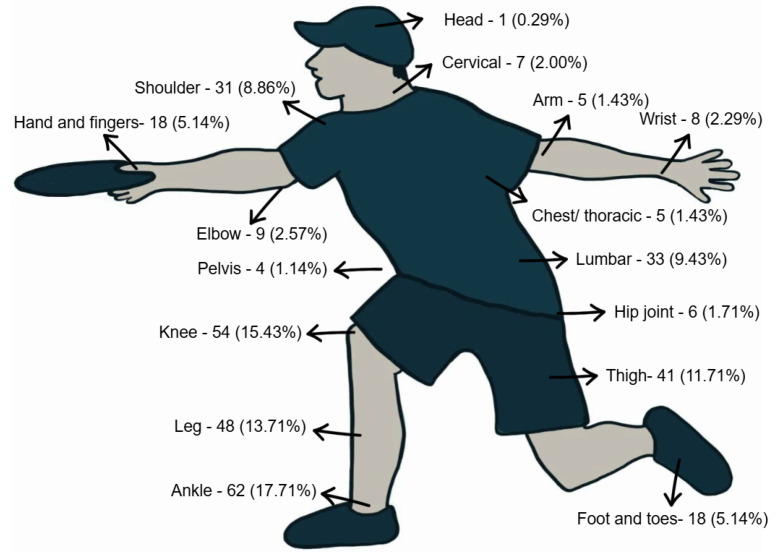
Anatomical locations of injuries.

Figure 2 shows the anatomical locations of the injuries reported by the athletes in the last 12 months.

Considering the occurrence of injuries, 172 (49.14%) occurred during training sessions, 117 (33.43%) during competitions, 44 (12.57%) without an apparent cause, 13 (3.71%) during warm-up before training sessions, games, or competitions, and 4 (1.14%) occurred during stretching or cool-down after training sessions, games, or competitions.

Table 3 presents the injury mechanisms reported by athletes over the past 12 months. Athletes were allowed to report more than one mechanism contributing to a single injury.

Regarding injury treatment, most injuries (317; 90.57%) received some form of treatment. Concerning the type of treatment, 237 (38.85%) injuries were treated with physiotherapy, 174 (28.52%) with rest, 80 (13.11%) with immobilization, 58 (9.51%) with medication, 23 (3.77%) with complementary therapies such as acupuncture, Reiki, or Shiatsu, 21 (3.44%) with surgery, 13 (2.13%) with osteopathy, and 4 (0.66%) with other treatments not specified.

Regarding the athlete’s downtime due to injury, 46 (13.14%) injuries did not prevent athletes from continuing their training, in 16 (4.57%) injuries athletes had to interrupt their training for up to 2 days, in 42 (12%) injuries athletes had to interrupt their training between 3 and 7 days, in 59 (16.86%) injuries athletes had to interrupt between 8 and 14 days, in 79 (22.57%) injuries athletes had to interrupt between 15 and 30 days and 108 (30.86%) injuries athletes had to interrupt frisbee practice for more than 30 days..

In 162 (46.29%) injuries, athletes reported feeling no pain at the time of data collection and being fully recovered from the injury; in 89 (25.43%) injuries, athletes reported feeling no pain but were undergoing treatment; in 62 (17.71%) injuries, athletes reported feeling pain and were undergoing treatment; and in 37 (10.58%) injuries, athletes reported feeling pain but were not undergoing any type of treatment.

The overall performance of the multivariable logistic regression model was assessed using several goodness-of-fit indicators. The Omnibus test of model coefficients was not statistically significant (χ^2^(13) = 16.177, *p* = 0.240), indicating that the inclusion of the independent variables did not significantly improve the model compared to the null model. The model explained a small proportion of the variance in injury occurrence, with a Cox & Snell R^2^ of 0.033 and a Nagelkerke R^2^ of 0.044. Nevertheless, the Hosmer–Lemeshow goodness-of-fit test suggested an adequate fit between observed and predicted values (χ^2^(8) = 6.667, *p* = 0.573). The contingency table further demonstrated a reasonable agreement between observed and expected frequencies across risk deciles. Overall, although the model showed acceptable calibration, its explanatory power was limited.

Table 4 presents the crude and adjusted associations between injury occurrence in the last 12 months and non-modifiable risk factors as well as frisbee practice characteristics. In the adjusted model, only years of frisbee practice remained significantly associated with injury occurrence, with players practicing for equal or more than 11 years of practice showing higher odds of injury compared to those until 10 years (OR = 1.84; 95% CI: 1.16–2.91; *p* = 0.009). No significant associations were observed for sex, age group, participation in another sport, BMI, weekly training frequency, or training duration (*p* > 0.05). Similarly, training surface and playing function were not significantly associated with injury occurrence in either crude or adjusted analyses, although most categories showed odds ratios above 1.

## 4. Discussion

This study evaluated musculoskeletal injuries among athletes who participated in the World Beach Ultimate Championship (WBUC), the most prestigious international beach ultimate club tournament, organized by the World Flying Disc Federation (WFDF), which brings together the top club teams worldwide. The findings revealed a high prevalence of injuries within the sample analyzed, with 81% of athletes reporting at least one injury throughout their frisbee practice, totalling 1685 injuries. In the past 12 months, 44% of athletes reported at least one injury, accounting for 358 injuries.

Similar findings were reported by Muramoto et al. [9], who observed that 49.1% of 116 athletes reported sustaining an injury in the previous year (2022), based on data from the Japanese University Athletic Association. Likewise, Pang et al. [10] assessed 59 ultimate frisbee athletes in Hong Kong in 2019 and found an injury prevalence of 62.7%. Khoo et al. [11] reported that 43 (98%) players sustained an ultimate-related injury throughout their practice, and 26 (63%) players were injured during the 2019 season.

Yen et al. [12] analyzed injuries leading to game stoppages over three days of the 2007 Ultimate Players Association College Championships in Ohio, USA (86 games), recording 107 injury timeouts, corresponding to an incidence rate of 1.66 per 1000 athlete-exposures during elimination rounds. In our study, the injury rate was 0.67 injuries per 1000 h of frisbee training. Pang et al. [10] reported 54 injuries over 9412 athlete exposures, resulting in an incidence rate of 5.74 per 1000 athlete exposures.

These differences should be interpreted with caution due to important methodological and contextual variations between studies. Yen et al. [12] reported an incidence of 1.66 injuries per 1000 athlete exposures during a 3-day tournament, considering only injuries that resulted in game stoppage. Similarly, Pang et al. [10] found a higher incidence rate based on competition-related exposures and considering the 2019 season. In contrast, the present study considered only training exposure over a longer period, which may partly explain the lower injury rate observed.

Additionally, the use of different exposure metrics (athlete exposures vs. hours of training) limits direct comparison between studies, as athlete exposures do not account for the duration or intensity of participation. Competition settings are also typically associated with higher physical and psychological demands, which may increase injury risk compared to training contexts.

Additionally, differences in injury definitions and data collection methods may have contributed to the variability in reported rates. For instance, restricting injury registration to events causing game interruption, as in Yen et al. [12], may underestimate less severe injuries, whereas other methodologies may capture a broader spectrum of injury severity.

A systematic review conducted by Pulido and Lystad [2], which included 11 studies up to 2020, reported that injury incidence rates ranged from 0.4 to 84.9 injuries per 1000 athlete exposures. Lifetime injury prevalence reached 100% in two of the included studies.

Akinbola et al. [13] compared injuries in ultimate frisbee with those in 32 other collegiate sports and artistic activities over a 12-year period (2000–2012), reporting that ultimate accounted for 143 (31%) of the 461 injuries recorded across all club sports, second only to rugby, which accounted for 156 cases (33.8%).

In our study, the most frequent injury types reported in the past 12 months were muscle strain (19%), followed by sprain (13%), tendinopathy (11%), and ligament injury (10%). Similarly, the systematic review by Pulido and Lystad [2] identified muscle injuries (range: 24.2–36.9%) and sprains (range: 7.6–49.1%) as the most common injury types. In our sample, muscle strains occurred predominantly in the lower leg and thigh. Khoo et al. [11] also identified thigh muscle strain as the most frequent injury type, followed by ankle sprains, both in the pre-season and post-season.

A sprain is a specific ligament injury that occurs when a joint is forced beyond its normal range of motion. The injury mechanism typically involves movements or forces such as ankle inversion, which is the most common, an awkward landing, or a sudden change in direction—one of the most frequently reported mechanisms in our study. The most commonly reported sprain among athletes occurred at the ankle.

Tendinopathy reported by athletes in this study occurred predominantly at the knee joint, as did ligament injuries. Muramoto et al. [9] also found ligament injuries (n = 78) and contusions (n = 57) to be the most common injury types.

Lazar et al. [14] specifically assessed the lifetime prevalence of concussions in ultimate frisbee among 787 players in the United States, reporting that 26.58% of men and 24.79% of women experienced at least one concussion. In contrast, no cases of concussion were reported in the present study.

The most frequently reported anatomical injury locations over the past 12 months were the ankle (18%), knee (15%), lower leg (14%), thigh (12%), lumbar spine (9%), and shoulder (9%). Most injuries involved the lower limbs. Similarly, Pulido and Lystad [2] identified the knee (19.5–39.7%), thigh (11.9–31.9%), and ankle (15.5–30.1%) as the most affected anatomical regions.

Yen et al. [12] also reported lower limb injuries as the most frequent, accounting for 53% in male teams and 51% in female teams. Comparable findings were reported by Khoo et al. [11], who observed a high prevalence of lower limb injuries (43 in pre-season and 23 in post-season) among elite ultimate players, with the most common injuries involving the hamstrings (14), ankle (13), anterior cruciate ligament (8), shoulder (14), and back (11).

Pang et al. [10] reported that 61% of injuries occurred in the lower limbs, with the foot and ankle (27.8%), knee (24.1%), lumbar spine (14.8%), and hand and wrist (9.3%) being the most affected areas. Muramoto et al. [9] identified the ankle, thigh, knee, and wrist as the most common injury sites, while Akinbola et al. [13] reported the knee (35%), foot/ankle (23.1%), and lower back/flank, hamstring, and shin/Achilles tendon injuries (7.7% each) as the most frequent.

The high frequency of jumping, rapid changes in direction, and pivoting during frisbee training and competition may explain the predominance of lower limb injuries [9,12].

The most reported injury mechanisms were repetitive movements (22%), changes in direction (16%), sprinting (15%), and collision with another player (13%). As injuries could be caused by more than one mechanism, athletes were allowed to report multiple contributing factors. In ultimate frisbee, intermittent running and sudden jumps may increase cardiovascular load, contributing to fatigue and consequently increasing the risk of non-contact injuries [5].

During training sessions and matches, directional changes involving “V”, “L”, or zig-zag movements are highly frequent, as well as sprinting to create separation or defend opponents. Palmer quantified the physical demands of elite women’s ultimate frisbee matches during an Australian national team tournament and reported that players performed 57 ± 27 high-speed runs and 45 ± 20 accelerations per match, indicating a very high proportion of sprinting activity [15].

Although diving actions (layouts)—where players launch themselves to catch or intercept the disc—are common in ultimate frisbee, this mechanism accounted for only 2% of injuries in the present study. However, such actions may result in strong player contact, which aligns with the finding that collisions with other athletes were among the most frequent injury mechanisms. Additionally, close marking and shadowing of opponents may further contribute to player-to-player contact [15].

Ultimate frisbee is officially a non-contact sport; however, accidental collisions do occur and are considered rule violations, resulting in a foul against the infringing team [15]. Unlike other team sports such as basketball or football, the findings of this study suggest that this classification may be questionable. Pulido and Lystad [2] reported that the most common injury mechanism was direct contact with another player (30.5–39.4%). Similarly, Yen et al. [12] found that most injuries leading to game stoppages were associated with contact and falls.

Muramoto et al. [9], however, reported that non-contact injuries were more prevalent than contact injuries (*p* < 0.01). Pang et al. [10] found that 15.8% of injuries resulted from collisions with another player, while other common mechanisms included cutting (23.7%), jumping (19.6%), pivoting (11.3%), stepping on uneven surfaces (11.3%), and falls (10.5%). In our study, 21% of athletes reported experiencing abrasions in the past 12 months.

In the present study, most injuries (49%) occurred during training sessions, while 33% occurred during competitions. Divergent findings were reported in the systematic review by Pulido and Lystad [2], where two studies indicated that the risk of injury was significantly higher during competitions than during training. Similarly, Pang et al. [10] reported that the risk of injury during training was three times lower than during tournaments (*p* < 0.01).

The higher risk of injury during competitions may be partly explained by increased physical demands, including faster running speeds and more frequent changes in direction, leading to greater mechanical load on muscles, joints, and ligaments. On top of that, athletes may perform diving actions (layouts) more frequently, attempt riskier plays, and ignore signs of fatigue or pain due to competitive demands. Official matches also tend to involve greater physical contact and more intense contests for the disc, with increased defensive pressure, thereby elevating the risk of collisions. Athletes often play multiple matches in a short period, reducing recovery time and contributing to cumulative fatigue. Field surface variability may also play a role; in the present study, the competition was held on sand, whereas most athletes trained on natural or artificial grass but frequently competed on both natural grass and sand.

The sand surface may influence movement biomechanics and external load, which could potentially affect injury patterns when compared to firmer surfaces such as grass or artificial turf. Sand typically increases energy expenditure, reduces impact forces, and alters movement patterns, which may affect both the type and frequency of injuries observed. Future research should aim to compare injury epidemiology across surfaces to better understand these differences.

Within this study, most injuries resulted in time loss exceeding 30 days (31%), which is consistent with the findings of Khoo et al. [11], where 27% of athletes were absent for one month or longer. Time loss due to injury may contribute to the development of mental health symptoms, such as anxiety and depression, negatively impacting psychological well-being and athletic career continuity [16].

The intrinsic risk factors analyzed in this study did not show statistically significant associations, which is consistent with the findings of Muramoto et al. [9]. Nevertheless, a non-significant trend towards a higher injury occurrence among male athletes was observed. Similar findings were reported by Khoo et al. [11], who found no differences between sexes in previous injury prevalence but observed a higher prevalence of injuries among men (72%) during the 2019 season and 100% during the pre-season. Pang et al. [10] reported a similar incidence between males and females, although without statistical significance (*p* = 0.63). In contrast, two studies reported a higher proportion of injuries among female athletes [12,13], with Akinbola et al. [13] indicating that women accounted for 71% of injuries in ultimate, compared to 29% in men (*p* < 0.001).

The only risk factor that showed statistical significance in the present study was years of practice, with athletes who had been practicing frisbee for more than 10 years demonstrating a higher risk of injury. This finding may be explained by greater cumulative exposure to training and competition, particularly due to overload and overuse resulting from repeated physical stress on the same anatomical structures. Notably, the most frequently reported injury mechanism in this study was repetitive movements, and the mean duration of practice among participants was 13 years. Jayanthi et al. [17] demonstrated an association between increased injury risk and higher levels of sports specialization, supporting the notion that greater specialization over time is linked to a higher risk of overuse injuries. Overall, these findings suggest a potential association between greater playing experience and increased injury risk, although this should be interpreted cautiously given the cross-sectional nature of the data and the lack of adjustment for potential confounders such as cumulative training load and previous injury history.

Nonetheless, this study has several limitations. Data collection was based on self-reported injuries, which may introduce recall bias. This is particularly relevant for injuries occurring earlier in the 12-month recall period, where underreporting of minor injuries and overreporting of more severe or memorable events may have occurred. Therefore, the magnitude of injury prevalence may be subject to both underestimation and overestimation depending on injury severity and timing.

Injuries were not clinically verified by healthcare professionals. Although definitions were provided in the questionnaire and researchers were available to clarify doubts, differences in individual perception may have occurred, as well as a tendency to overestimate or underestimate reported events, potentially affecting response consistency. The absence of a medical diagnosis may have led to misclassification of injury status and type, limiting diagnostic accuracy. Future studies, including clinical confirmation of reported injuries by healthcare professionals, are warranted; however, this may limit the ability to recruit a representative sample.

Exposure was measured using self-reported training frequency and duration rather than directly recorded athlete exposure hours. This limits the precision of the calculated injury incidence rate and may reduce comparability with studies using more objective exposure metrics.

Moreover, the use of convenience sampling represents an important limitation, as participants were recruited based on accessibility rather than through random selection. This approach increases the risk of selection bias, as individuals with prior injury experience or greater interest in the topic may have been more likely to participate. As a result, certain subgroups may be overrepresented, while others are underrepresented, limiting the extent to which the findings can be generalized to the broader population. Finally, the lack of rigorous control of potential confounding factors may have influenced the observed associations, and results should therefore be interpreted with caution.

Some of the associations reported in this study should be considered exploratory rather than causal. While the observed patterns provide useful descriptive insights into injury epidemiology in ultimate frisbee athletes, they should not be interpreted as definitive causal relationships.

The multivariable logistic regression model demonstrated limited explanatory capacity, as indicated by low pseudo-R^2^ values (Cox & Snell R^2^ = 0.033; Nagelkerke R^2^ = 0.044) and a non-significant overall model fit (Omnibus test *p* = 0.240). These results suggest that the included variables explain only a small proportion of injury occurrence, highlighting the multifactorial nature of sports injuries.

This limited explanatory power indicates that relevant confounding factors may not have been captured in the present analysis. Mainly, variables such as training load, previous injury history, competition level, and recovery strategies were not included and may play an important role in injury risk. The absence of these variables may partially explain the weak associations observed and the overall low predictive performance of the model.

## 5. Conclusions

The data from this study revealed a high prevalence of musculoskeletal injuries among the athletes who comprised the sample, particularly in the lower limbs, with muscle strains and sprains being the most common type of injuries.

Years of practice were the only factor significantly associated with injury occurrence (≥11 years; OR = 1.84). However, this result should be interpreted with caution, as the study design does not allow causal or predictive conclusions. The observed association may reflect greater cumulative exposure rather than a direct effect of years of practice.

These findings highlight the importance of implementing practical injury prevention strategies, such as appropriate load management, strength and neuromuscular training, and improved movement control. Further research, particularly using prospective designs and clinical assessment of injuries, would be valuable to better understand injury patterns and support safer participation in the sport.

## Figures and Tables

**Table 1:** Characteristics of frisbee practice.

Quantitative Variables	Minimum–Maximum (Mean ± Standard Deviation)
Years of frisbee practice	1–40 years (13.01 ± 7.20)
Weekly training frequency	2–7 times per week (2.96 ± 1.15)
Weekly training duration	1–30 h per week (7.16 ± 3.72)
Qualitative variables	n (%)
Practice another sport at least twice a week.	
Yes	286 (59.09%)
No	198 (40.91%)
Type of training surface	
Natural grass	238 (49.17)
Artificial grass	113 (23.35%)
Sand	96 (19.83%)
Indoor	37 (7.64%)
Type of competition surface	
Natural grass	226 (46.69%)
Sand	171 (35.33%)
Artificial grass	58 (11.98%)
Indoor	29 (5.99%)
Performing a warm-up before training and/or competition Duration varied between 5 and 90 min (23.91 ± 12.58)	
Yes	477 (98.55%)
No	7 (1.45%)
Cool-down exercise after training and/or competition Duration varied between 1 and 30 min (11.69 ± 5.78)	
Yes	222 (45.87%)
No	262 (54.13%)
Presence of a physical trainer on the team	
Yes	212 (43.80%)
No	272 (56.20%)
Player Role	
O-Line Cutter	133 (27.48%)
O-Line Handler	122 (25.21%)
D-Line Cutter	117 (24.17%)
D-Line Handler	112 (23.14%)
Competition Divisions	
Mixed	107 (22.110%)
Open	106 (21.90%)
Women’s	90 (18.60%)
Masters Mixed	61 (12.60%)
Masters Open	26 (5.37%)
Great Grand Masters Open	23 (4.75%)
Grand Masters Open	22 (4.55%)
Masters Women	21 (4.34%)
Grand Masters Mixed	16 (3.31%)
Grand Masters Women	12 (2.48%)

**Table 2:** Association between the type and location of injuries.

**Injury Type**	**Injury Location**	**n**	**%**
Muscle strain	Back/Chest	1	
	Shoulder	4	
	Arm	1	
	Elbow	2	
	Wrist	1	
	Hand and fingers	2	
	Pelvis	3	
	Thigh	20	
	Knee	2	
	Leg	25	
	Ankle	3	
	Feet and toes	2	
	Total	66	18.86%
Sprain	Wrist	5	
	Hand and fingers	2	
	Knee	4	
	Ankle	35	
	Feet and toes	1	
	Total	47	13.43%
Tendinopathy	Shoulder	10	
	Wrist	1	
	Hand and fingers	2	
	Hip joint	2	
	Knee	12	
	Ankle	8	
	Feet and toes	3	
	Total	38	10.86%
Ligament injury	Shoulder	6	
	Wrist	1	
	Hand and fingers	1	
	Hip joint	2	
	Knee	17	
	Ankle	8	
	Total	35	10.00%
Fracture	Skull/head	1	
	Back/Chest	1	
	Arm	1	
	Forearm	1	
	Hand and fingers	8	
	Leg	2	
	Ankle	3	
	Feet and toes	9	
	Total	26	7.43%
Muscle tear	Shoulder	1	
	Thigh	13	
	Leg	11	
	Ankle	1	
	Total	26	7.43%
Traumatic muscle contusion	Arm	2	
	Thigh	6	
	Knee	3	
	Leg	8	
	Ankle	1	
	Total	20	5.71%
Dislocation	Lumbar spine	1	
	Shoulder	10	
	Hand and fingers	3	
	Ankle	1	
	Feet and toes	1	
	Total	16	4.57%
Cartilage injury	Hip joint	2	
	Knee	5	
	Ankle	2	
	Total	9	2.57%
Meniscus injury	Knee	8	
	Total	8	2.29%
Epicondylitis	Elbow	7	
	Total	7	2.00%
Plantar fasciitis	Feet and toes	2	
	Total	2	0.57%
Low back pain	Lumbar spine	32	
	Total	32	9.14%
Thoracic pain	Back/Chest	3	
	Total	3	0.86%
Neck pain	Cervical spine	7	
	Total	7	2.00%
Non-specific pain	Arm	1	
	Pelvis	1	
	Thigh	2	
	Knee	3	
	Leg	1	
	Total	8	2.29%
Total		350	100%

**Table 3:** Mechanisms of injury.

Injury Mechanisms	n	%
Repeated movements	84	22.11
Changes in direction	62	16.32
Sprint	57	15.00
Impact with another athlete	48	12.63
Landing	36	9.47
Fall	24	6.32
Twisting motion	20	5.26
Decelerations	13	3.42
Direct impact (Frisbee)	13	3.42
Diving	9	2.37
Receiving the disc (Standing)	4	1.05
Other	10	2.63
Total	380	100

**Table 4:** Relationship between injury occurrence in the last 12 months and non-modifiable risk factors and characteristics of frisbee practice.

Variables	Odds Ratio—Crude (95%); *p*-Value	Odds Ratio—Adjusted (95%); *p*-Value
Sex (female *) male	1.15 (0.80)1.66; 0.447	1.04 (0.791–1.52); 0.851
Age group (until 32 years old *) ≥ 33 years old	1.43 (0.99–2.04); 0.55	1.01 (0.638–1.591); 0.972
Practice of another modality (yes *) no	1.09 (0.75–1.56); 0.659	1.12 (0.77–1.65); 0.554
BMI (until 24.99 Kg/m^2^ *) ≥ 25 Kg/m^2^	1.44 (0.89–2.33); 0.137	1.32 (0.79–2.18); 0.286
Years of frisbee practice (until 10 years *) ≥ 11 years	1.79 (1.24–2.59); 0.002	1.84 (1.16–2.91); 0.009
Weekly training frequency (until 2 times *) ≥ 3 times	1.16 (0.81–1.67); 0.410	1.19 (0.79–1.78); 0.407
Weekly training duration (until 6 h *) ≥ 7 h	1.02 (0.71–1.46); 0.912	1.06 (0.71–1.59); 0.773
Training surface (natural grass *)		
Indoor	1.40 (0.66–2.99); 0.385	1.25 (0.57–2.74); 0.584
Artificial grass	1.39 (0.64–3.02); 0.406	1.19 (0.53–2.67); 0.669
Sand	1.21 (0.59–2.47); 0.598	1.09 (0.52–2.28); 0.828
Function performed in frisbee (O-Line Cutter *)		
O-Line Handler	1.27 (0.76–2.12); 0.369	1.37 (0.80–2.33); 0.254
D-Line Cutter	1.42 (0.84–2.39); 0.187	1.39 (0.82–2.37); 0.226
D-Line Handler	1.03 (0.63–1.70); 0.904	1.09 (0.65–1.83); 0.738

* Class reference; CI: Confidence interval.

## Data Availability

The data presented in this study are available on reasonable request from the corresponding author. The data presented in this study are not publicly available due to privacy and ethical restrictions.
